# Interkingdom Comparison of Threonine Metabolism for Stem Cell Maintenance in Plants and Animals

**DOI:** 10.3389/fcell.2021.672545

**Published:** 2021-09-07

**Authors:** Debee Prasad Sahoo, Lon J. Van Winkle, Rocío I. Díaz de la Garza, Joseph G. Dubrovsky

**Affiliations:** ^1^Departamento de Biología Molecular de Plantas, Instituto de Biotecnología, Universidad Nacional Autónoma de México, Cuernavaca, Mexico; ^2^Department of Biochemistry, Midwestern University, Downers Grove, IL, United States; ^3^Department of Medical Humanities, Rocky Vista University, Parker, CO, United States; ^4^Tecnologico de Monterrey, Escuela de Ingeniería y Ciencias, Monterrey, Mexico

**Keywords:** stem cells, threonine metabolism, threonine synthesis, threonine catabolism, embryonic stem cells, epigenetic modifications, root apical meristem, one-carbon metabolism

## Abstract

In multicellular organisms, tissue generation, maintenance, and homeostasis depend on stem cells. Cellular metabolic status is an essential component of different differentiated states, from stem to fully differentiated cells. Threonine (Thr) metabolism has emerged as a critical factor required to maintain pluripotent/multipotent stem cells in both plants and animals. Thus, both kingdoms conserved or converged upon this fundamental feature of stem cell function. Here, we examine similarities and differences in Thr metabolism-dependent mechanisms supporting stem cell maintenance in these two kingdoms. We then consider common features of Thr metabolism in stem cell maintenance and predict and speculate that some knowledge about Thr metabolism and its role in stem cell function in one kingdom may apply to the other. Finally, we outline future research directions to explore these hypotheses.

## Introduction

Stem cells are defined as “cells that have the ability to divide for indefinite periods in an undifferentiated state but retain the potential to give rise to specialized cells” (Boiani and Schöler, 2005). Cellular metabolic status is an essential component of different cell states, from completely undifferentiated stem cells to fully differentiated tissues and organs (e.g., Spyrou et al., 2019; Liu et al., 2020). Stem cells in animals can be of two main type, embryo derived, such as embryonic stem (ES) cells, and adult stem cells (Raff, 2003). The former are an excellent model system for the studies of stem cell properties as they maintain proliferation capacity and can be cultivated *in vitro* for years (Boiani and Schöler, 2005). Barlow (1978) introduced the concept of stemness to the plant world and considered that initial cells, giving rise to all tissue types, should be considered stem cells. This small group of cells operate in plants within meristems, a large group of cells with high proliferation activity that constitutes the transit-amplifying cell population (Scheres, 2007; Sanz et al., 2012; Choe and Lee, 2017). Meristems originate from embryonic shoot (plumule) and root (radicle) and remain fully functional during the postembryonic lives of plants. The meristems are known to be of two main types: apical meristems, formed at the tip of growing organs and involved in organ growth in length, and lateral (or secondary) meristems that permit growth in girth. Homeostasis of meristems strongly depends on activity of stem cells (that are also recognized as initial cells giving rise to various cell types). Stem cells form a niche which is defined as microenvironment that provides support and stimuli necessary to maintain stem cell properties (Schofield, 1978; Smith, 2006). Comparisons of stem cell niche organization between kingdoms are infrequent but useful (Laux, 2003; Scheres, 2007; Heidstra and Sabatini, 2014; Matos and Bergmann, 2014). They provide insights in understanding common mechanisms, such as organization of bifacial stem cell niches regulated by homeobox transcription factors in fish retina and plant cambium cells (Shi et al., 2017) or similarities in transcription factors involved in stomata cell lineage and muscle development (Matos and Bergmann, 2014).

Despite large evolutionary distance, there are common features of plant and animal stem cells which include in most cases the following: (1) stem cells are spatially associated with organizing cells or organizing centers; (2) at least one daughter of a stem cell maintains stem cell properties; (3) stem cell progeny can acquire identity of one or a number of cell types; (4) stem cells and their niche are surrounded by transit-amplifying cells; (5) the programs for cell differentiation are strongly repressed in stem cells; and (6) stem cell maintenance is regulated by networks of transcription factors, and these networks are kingdom specific (Laux, 2003; Sablowski, 2004; Smith, 2006; Scheres, 2007; Matos and Bergmann, 2014; Kilberg et al., 2016). Kingdom-specific networks of transcription factors for stem cell maintenance (Pysh et al., 1999; Nole-Wilson et al., 2005; Lee et al., 2008; Nardmann et al., 2009) could mean that no common ancestral mechanisms exist to control stem cell properties in plants and animals.

However, recent studies support the notion that metabolism-dependent mechanisms of development could be ancestral. A growing body of information shows that such a mechanism is of paramount importance for both plant (Koch, 2004; Mehrshahi et al., 2010; Ljung, 2013) and animal (Miyazawa and Aulehla, 2018) development. Here, we identify an amino acid, L-threonine (Thr), as a common and likely ancestral factor involved in maintenance of stem cell properties in plants and animals, and we discuss similarities and differences in this mechanism of stem cell maintenance in the two kingdoms. Finally, we predict and speculate about what we can learn or deduce concerning Thr signaling and metabolism in one kingdom, based on what we know about these mechanisms in the other kingdom.

## Mammalian Embryonic Stem and Their Progenitor Cells in Early Embryos Need Thr Signaling and Metabolism to Remain Pluripotent and Proliferate

Several days after sperm and egg cells unite, distinct cell types appear in preimplantation mammalian blastocysts, namely, the trophectoderm and inner cell mass (ICM). A single layer of cells on the surface of embryos forms the trophectoderm, which makes contact with the uterine epithelium during implantation. All other mammalian tissues arise from the ICM. ES cells are derived from and serve as a model for the ICM (Van Winkle and Ryznar, 2019a,b).

Threonine is critical for ES cell maintenance. If Thr is not supplied in their culture medium, murine (mES) and probably bovine ES cells cannot proliferate nor do they remain undifferentiated (Wang et al., 2009; Najafzadeh et al., 2018). In these ES cells, Thr is converted to glycine and acetyl CoA by threonine dehydrogenase (TDH). In addition to transcription of its genes, posttranscriptional and posttranslational effectors regulate TDH activity (Han et al., 2013). The glycine and acetyl CoA generated by TDH are required precursors that help to regulate epigenetic modifications, nucleotide biosynthesis, and mitochondrial free energy conversions in these cells (Shyh-Chang et al., 2013; Tian et al., 2019).

While mES cells are exposed to an optimum concentration of Thr in their culture medium, mES progenitor cells in the ICM must obtain their amino acids *via* the blastocyst trophectoderm. The ICM is in direct contact with some trophoblast cells and likely receives amino acids from them, while other ICM cells are in contact with the fluid-filled cavity of blastocysts (blastocoelic fluid) and may obtain amino acids directly from the blastocoel (Gardner, 2000). Each of more than 12 Na^+^-dependent and 7 Na^+^-independent amino acid transport system activities in the blastocyst trophectoderm helps to provide amino acids to their mES progenitor cells (Van Winkle, 2001; Van Winkle and Ryznar, 2019a). However, none of these systems transports Thr efficiently (Van Winkle, 2001; Van Winkle and Ryznar, 2019a). Hence, another mechanism likely helps blastocysts provide Thr to their ICMs in order to maintain their undifferentiated, proliferating state.

Despite the absence of transporters with relatively low *K*_*m*_ values for Thr, mouse blastocysts accumulate Thr as they approach implantation (Martin et al., 2003; Van Winkle and Ryznar, 2019a). Even blastocysts developing *in vitro* in the absence of amino acids accumulate Thr as they near the time that they could attach to the uterus. Since animals cannot synthesize Thr, trophoblast cells likely hydrolyze protein from the medium *in vitro*, or uterine fluid *in vivo*, to produce Thr and supply it to the ICM (Van Winkle and Ryznar, 2019a). The trophoblast is physiologically poised to perform this function since it exhibits active pinocytosis (Sun et al., 2014).

Mouse ES and their progenitor cells then take up this free Thr *via* at least three obligate exchange amino acid transporters in their cell membranes. The Na^+^-dependent Alanine, Serine, Cysteine as well as Thr-preferring amino acid transporters, ASCT1 and ASCT2, are abundant in these cells, as is the Na^+^-independent Thr transporter, LAT2, or another system L transporters (Formisano and Van Winkle, 2016). In addition to providing substrates for epigenetic modification and other metabolic processes, Thr fosters mES cell proliferation and pluripotency more directly through its biomembrane transport (Ryu and Han, 2011).

Threonine promotes mammalian target of rapamycin (mTOR)-mediated signaling by fostering cMyc expression in mES cells. This Thr-initiated signaling cannot occur if lipid rafts containing Thr transporter(s) are disrupted (Ryu and Han, 2011). The Thr analog, 3-hydroxynorvaline (3-HNV), likely blocks mES cell proliferation by competing for Thr transport (Formisano and Van Winkle, 2016) as well as by inhibiting TDH and Thr catabolism (Wang et al., 2009; see below). Similarly, this signaling and metabolism could help maintain pluripotency and proliferation in the ICMs of blastocysts. As discussed above, contrary to other amino acids, Thr accumulates in mouse blastocysts as they approach implantation in the uterus (Martin et al., 2003; Van Winkle and Ryznar, 2019a).

Because mES and their progenitor cells need signaling both due to Thr transport and its metabolism, it was not surprising to find that 3-HNV blocks not only mES but also hES cell growth (Van Winkle et al., 2014). Historically, 3-HNV has been used as a specific inhibitor of TDH in mES cells (Wang et al., 2009). Moreover, both 3-HNV and the TDH inhibitor, quinazolinecarboxamide1 (Qc1; Alexander et al., 2011), stop mouse blastocyst development, and they abolish or slow bovine blastocyst development, respectively (Wang et al., 2009; Najafzadeh et al., 2018). The Qc1 inhibitor kills mES cells due to strong and specific inhibition of TDH (Alexander et al., 2011). Because hES cells express inactive forms of TDH, however, they cannot catabolize Thr to produce the substrates they need for epigenetic histone modification and maintenance of an undifferentiated state. Nevertheless, 3-HNV could block hES cell proliferation through inhibition of Thr transport and signaling (Van Winkle et al., 2014).

It was suggested that 3-HNV slows proliferation of cells lacking TDH activity by incorporation into protein in place of Thr (Najafzadeh, 2018). Interestingly, proliferation of mouse cell lines (other than mES cells) and HeLa cells (a human cell line) are not inhibited by 3-HNV (Wang et al., 2009), so 3-HNV does not block cell growth owing to incorporation into protein. Rescue of 3-HNV-inhibited hES proliferation by excess Thr is consistent with the theory that hES (and mES) cells need Thr for signaling separate from (and in addition to) Thr catabolism for epigenetic histone modifications (Van Winkle et al., 2014).

### TDH and the Glycine Cleavage System Maintain ES Cells

**FIGURE 1:**
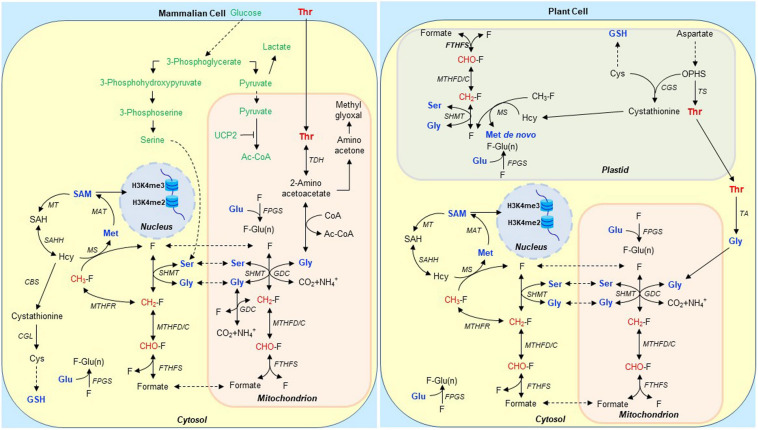
Schematic representation of Thr and 1C metabolism in mammalian and heterotrophic plant cells involved in stem cell maintenance. We propose that Thr is taken up by a subpopulation of perinuclear mitochondria specialized to metabolize it to formate in mammalian ES cells. The mitochondria then release the formate to a compartment used specifically for H3K4me di- and tri-methylation *via S*-adenosyl methionine (SAM) 1-carbon (1C)-dependent metabolism (Van Winkle and Ryznar, 2019b). When the GDC is inhibited, it is unable to complete Thr catabolism. Thus, H3K4me3 formation is suppressed, and the 2-amino acetoacetate produced from Thr is instead converted to methylglyoxal which fosters ES cell senescence (Kang et al., 2019; Tian et al., 2019). Expression of enzymes in the serine synthesis pathway in TDH-deficient hES cells is up-regulated likely to circumvent the need for Thr metabolism *via* TDH (Tian et al., 2019). This specific pathway in hES cells is shown in green in mammalian cell; a similar pathway in plant cell is not shown. In most mammalian ES cells, serine derived from glycolytic intermediates acts as a major source of 1C units (Tibbetts and Appling, 2010). In heterotrophic plant cells, Thr is synthesized in undifferentiated and non-photosynthetic plastids. Folates (F) and their polyglutamylated forms are accumulated in plastids, mitochondria, and the cytosol. Polyglutamylated folates [F-Glu(n)] are the preferred cofactors for 1C enzymes, and FPGS mediates the addition of Glu residues to folate derivatives. Thr synthesis and polyglutamylated folates are critical for stem cell maintenance (Reyes-Hernández et al., 2014; Reyes-Hernández et al., 2019; see also Figure 2). Groups marked in red are 1C units derived from Thr, Ser, Gly, and formate, which are eventually used for H3K4me2 and H3K4me3 methylations. Abbreviations: Ac-CoA, acetyl coenzyme A; CBS, cystathionine β-synthase; CGL, cystathionine γ-lyase; CGS, cystathionine γ-synthase; FTHFS, 10-formyltetrahydrofolate synthetase; FPGS, folylpolyglutamate synthetase; GDC, Gly decarboxylase complex; GSH, glutathione; Hcy, homocysteine; Met, methionine; MAT, Met adenosyltransferase; MS, Met synthase; MTHFC, 5,10-methenyl-THF cyclohydrolase; MT, methyltransferase; MTHFD, 5,10-methylene-THF dehydrogenase; MTHFR, 5,10-methylene-THF reductase; OPHS, O-phosphohomoserine; SAH, *S*-adenosylhomocysteine; SAHH, *S*-adenosyl-homocysteine hydrolase; SAM, *S*-adenosylmethionine; SHMT, serine hydroxymethyltransferase; TA, Thr aldolase; TCA cycle, tricarboxylic acid cycle; TDH, Thr dehydrogenase; THF, tetrahydrofolate; TS, Thr synthase; and UCP2, uncoupling protein 2.

While most mammalian ES and their progenitor cells likely need TDH to produce acetyl CoA and glycine and remain pluripotent, hES cells express inactive forms of this enzyme and do not require it (Edgar, 2002). Likely to circumvent the need for Thr metabolism *via* TDH, enzymes in the serine synthesis pathway (Figure 1) are greatly up-regulated in hES cells but not in mES or mouse-induced pluripotent stem cells (Tian et al., 2019). The resultant serine is then converted to glycine. Subsequently, the Gly decarboxylase complex (GDC) functions to maintain proliferation in all ES cell types studied so far, and its activity must be up-regulated in order to form induced murine and human pluripotent stem cells (Kang et al., 2019; Tian et al., 2019). This system metabolizes glycine away from methylglyoxal formation (Figure 1) which would otherwise foster senescence of stem cells (Kang et al., 2019; Tian et al., 2019). Instead, the use of glycine for 1-carbon (1C) metabolism promotes specific epigenetic histone modifications needed to keep hES and mES cells pluripotent (Ang et al., 2011; Kilberg et al., 2016; Chen and Wang, 2019; Van Winkle and Ryznar, 2019b). Specialization of mitochondria for the latter metabolic processes is facilitated by the nearly exclusive reliance of ES cells on glycolysis for ATP generation (Spyrou et al., 2019) owing to shunting pyruvate away from mitochondria by uncoupling protein 2 (Zhang et al., 2011; Chakrabarty and Chandel, 2021). Tryptophan metabolism also regulates proliferation of human pluripotent stem cells, thus, also likely helping the cells to obviate a need for Thr catabolism (Someya et al., 2021).

**FIGURE 2:**
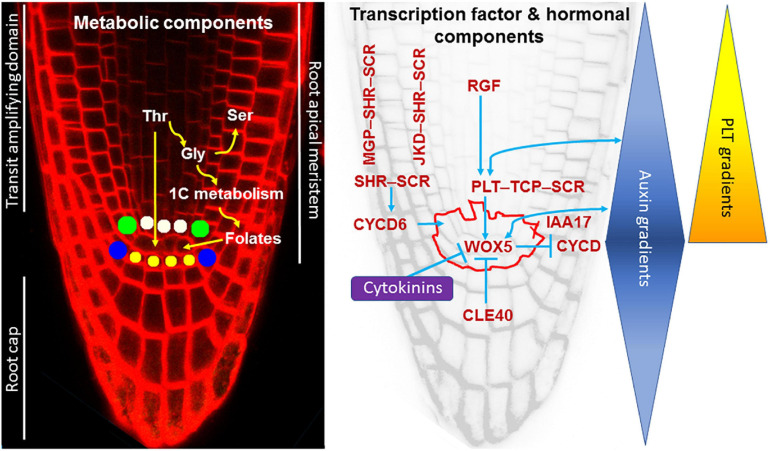
Organization of the root apical meristem (RAM) and control of stem cell function. Left, *A. thaliana* root apex and metabolic components of the stem cell maintenance. Stem cells surrounding the quiescent center (QC) are the following: yellow stem cells form columella; blue stem cells give rise to the lateral root cap and epidermis; green stem cells give rise to ground tissues (cortex and endodermis) and cells marked with white give rise to central cylinder tissues. Root apex includes the RAM and the root cap. Transit-amplifying domain includes derivatives of the stem cells. Right, a gene regulatory network for RAM maintenance and its interaction with plant hormones, auxin, and cytokinins. Outlined area represents the QC and stem cells. See the text for details. A member of the AUX/IAA family of transcriptional regulators, IAA17, is an intermediate between auxin and WOX5 (Tian et al., 2014). Other details can be found in Shimotohno and Scheres (2019). Auxin and PLT gradients have a maximum in the QC.

### mES Cells Require Thr Catabolism for Specific Histone Modifications

If Thr is absent from their culture medium, mES cells begin to differentiate (Wang et al., 2009). These cells normally catabolize Thr to glycine and acetyl CoA, and the glycine cleavage system converts glycine to 1C units needed as substrates to regulate epigenetic modifications (Jog et al., 2017). When Thr is not supplied in the medium, DNA and histone methylation continue normally except for di- and tri-methylation of lysine (Lys) residue 4 of histone H3 (H3K4me3), which slows dramatically and selectively (Shyh-Chang et al., 2013). To remain pluripotent and proliferate, human as well as mouse ES cells need H3K4me3 (Ang et al., 2011; Kilberg et al., 2016; Chen and Wang, 2019; Van Winkle and Ryznar, 2019b). Thus, Thr taken up from the medium seems somehow to be metabolized selectively to provide 1C units for H3K4 methylation in mES cells. However, by what mechanism might this selection occur?

To illustrate one possibility, leucine taken up by the trophectoderm of preimplantation blastocysts triggers mTOR signaling. This signaling leads to trophoblast motility and penetration of the uterine epithelium (Van Winkle et al., 2006). Only leucine uptake *via* the B^0,+^ amino acid transporter fosters this mTOR signaling. Trophoblast motility does not occur when leucine accumulates in these cells *via* other transporters in blastocysts (González et al., 2012). Amino acid transport system B^0,+^ either generates other signals needed to synergize with mTOR, or it selectively directs leucine to sites of mTOR signaling.

In mES cells, selective formation of H3K4me3 using Thr may be more complex. Perinuclear mitochondria in mES cells carry out Thr catabolism (Wanet et al., 2015; Lees et al., 2017; Woods, 2017). We suggest that a subpopulation of these organelles take up Thr selectively (Figure 1). They then metabolize Thr to formate, which is transported to the cytosol where it is converted to *S*-adenosyl methionine (SAM), methyl group donor required for H3K4 methylation in the nucleus (Scotti et al., 2013; Harvey, 2019). The formate from these mitochondria must be directed specifically to nuclear sites of H3K4 methylation, because 1C units originating from Thr, but not other sources, are used to methylate this H3 residue (Shyh-Chang et al., 2013). Glycine can also be used for this purpose in mES cells but only when an excess of it is supplied to the cells (Figure 1).

Alternately, one of at least three Thr transporters in the plasma membrane (Formisano and Van Winkle, 2016) could somehow direct Thr to the subpopulation of perinuclear mitochondria, much like the B^0,+^ transporter in the trophectoderm seems to direct leucine to sites of mTOR signaling (Van Winkle et al., 2006).

In summary, epigenetic DNA and histone modifications in ES and their progenitor cells in early embryos require membrane transport and compartmentalized metabolism of amino acids. One-carbon metabolites, produced specifically from Thr taken up from the culture medium, regulate H3 methylation in mES and probably bovine and other mammalian ES cells. ES cells require these 1-carbon units to produce H3K4me3 and remain undifferentiated. Further understanding of this regulation is essential to the study of all mammalian species (except TDH-deficient humans) because all adult tissues and organs arise from ES progenitor cells.

## Role of Thr Synthesis in Stem Cell Maintenance in Plants

In contrast to animals, where Thr is an essential amino acid, plants synthesize Thr within plastids. This Thr is important for plant metabolism and development. In this section, we consider how and where stem cells function in plants, and then we address the role of Thr synthesis for plant stem cell maintenance.

### Apical Meristem Organization and Stem Cell Control

Plants grow in length due to the activity of their meristems, located at the apices of the growing shoot and root. Due to rigid cell walls, plant cells do not move and grow symplastically (Sinnott, 1960; Erickson, 1986). At the very tip of the apical meristem, an organizer, a group of proliferatively quiescent cells, represents a stem cell niche, surrounded by initial or stem cells. Their descendants enter the transit-amplifying domain, which constitute the main body of the apical meristem. Thus, shoot and root growth result from cell production in the meristem and their subsequent enlargement. As the role of Thr till now is established only for the root apical meristem (RAM), we will focus mainly on its organization and function.

In the RAM, the organizer, called the quiescent center, QC (Clowes, 1956), behaves as a stem cell niche and maintains surrounding stem cells in an undifferentiated state (Van Den Berg et al., 1995, 1997). In a model plant, *Arabidopsis thaliana*, which develops a very thin, about 1/4 mm in diameter, root, the QC is a small group of cells (Figure 2). Thicker roots, such as in maize, which are about 1 mm in diameter, contain QCs composed of up to a thousand cells (Jiang et al., 2003). In *A. thaliana*, the stem (initial) cells located around the QC (Dolan et al., 1993) give rise to all tissue types (Figure 2). While cell cycle duration in the transit-amplifying domain in young *A. thaliana* seedlings, irrespective of accession, is on average 16.6 h (Zhukovskaya et al., 2018), proximal stem cells divide on average every 58.6 h (Rahni and Birnbaum, 2019).

The QC and stem cell identity and function depend on a complex gene regulatory network of transcription factors, peptides, and a closely linked hormonal regulation (reviewed in Drisch and Stahl, 2015; Shimotohno and Scheres, 2019; García-Gómez et al., 2020). Here, we only briefly outline the main components of this network which are critical for stem cell function. The first identified components of this network, SHORT ROOT (SHR) and SCARECROW (SCR) belong to the GRAS transcription factor family (Helariutta et al., 2000; Sabatini et al., 2003). Loss-of-function mutations in each of these genes (Benfey et al., 1993; Sabatini et al., 2003) among other defects, result in loss of stem cell activity, gradual disappearance of the transit-amplifying domain of the RAM, slough off the root cap and, thus, to complete RAM exhaustion (the phenomenon when all meristematic, including stem cells, become differentiated, and organ growth cannot be maintained). The APETALA2 transcription factors, PLETHORA, are also indispensable for stem cell function, and in *plt1plt2* double mutants, the RAM becomes exhausted (Aida et al., 2004; Galinha et al., 2007). Such a strong phenotype is not frequently found and even mutants in a homeobox transcription factor, *WUSCHEL RELATED HOMEOBOX 5* (*WOX5*), a gene that determines the QC identity, only columella stem cells become differentiated, but no RAM exhaustion takes place (Sarkar et al., 2007).

PLT proteins form a gradient along the transit-amplifying domain of the RAM with a maximum in the QC (Galinha et al., 2007; Mähönen et al., 2014) where PLT proteins restrict WOX5 expression domain to the QC (Santuari et al., 2016; Burkart et al., 2019). WOX5 promotes cell quiescence by direct transcriptional repression of *CYCLIN D* (*CYCD*; Forzani et al., 2014). Moreover, WOX5 promotes *PLT* expression (Ding and Friml, 2010; Burkart et al., 2019), creating a transcriptional feedback loop that reinforces quiescence of the QC cells. PLT1 and PLT3 transcriptionally activate WOX5 through binding to its promoter and, at the same time, PLT proteins interact indirectly with SCR, through class I members of the Teosinte-branched Cycloidea PCNA (TCP) proteins (Shimotohno et al., 2018). Although PLT3 alone can induce *WOX5* expression, this is significatively enhanced in the presence of TCP20 and SCR (Shimotohno et al., 2018), suggesting that PLT-TCP-SCR complexes directly regulate the promoter activity of *WOX5*. Therefore, PLT-TCP-SCR complexes promote WOX5 expression. SCR and SHR form heterodimers and both are critical not only for the QC identity but also for asymmetric cell division of ground tissue stem cells (Benfey et al., 1993; Di Laurenzio et al., 1996; Helariutta et al., 2000; Cui et al., 2007; Hirano et al., 2017). Here, cell division results from CYCD6 activity induced by SHR-SCR (Sozzani et al., 2010; Hirano et al., 2017). In addition, SHR-SCR heterodimer interacts with zinc finger transcription factors of the BIRD family members, e.g., JACKDAW (JKD) and MAGPIE (MGP), and together they control stem cell asymmetric division (Welch et al., 2007; Hirano et al., 2017; Long et al., 2017). This complex gene regulatory network involved in the QC and stem cell identities and activities operates in concert with some peptides and hormones. Auxin, which shows a concentration maximum in the QC (Sabatini et al., 1999; Blilou et al., 2005), is the main hormone involved in control of stem cell function; its synthesis and transport are under PLT control (Santuari et al., 2016). Auxin in turn affects PLT gene expression, so both factors form a positive feedback loop, though with a different time scale (Aida et al., 2004; Mähönen et al., 2014). *PLT* expression is also under control of tyrosine-sulfated peptides, ROOT MERISTEM GROWTH FACTORS (Matsuzaki et al., 2010). Auxin maximum in the QC and its graded distribution in the RAM, in addition to auxin transporters, are modulated by WOX5 (Ding and Friml, 2010; Tian et al., 2014) and SHR (Cruz-Ramírez et al., 2012). The main components of the regulatory network involved in stem cell activity in the RAM are shown in Figure 2.

### Role of Thr Synthesis in the Stem Cell Maintenance in Plants

In search of new regulatory pathways involved in the RAM maintenance, a forward genetic screen of a large population of ethylmethanesulfonate-mutagenized seeds permitted isolation of a mutant that showed stunted root growth and complete RAM exhaustion; the mutant was denominated *moots koom1* (*mko1*) that in Mayan means “short root” (Hernández-Barrera et al., 2011). The *mko1* phenotype was caused by a point mutation in *METHIONINE OVER-ACCUMULATOR 2* (*MTO2*, AT4G29840) encoding THREONINE SYNTHASE1 (TS1; Reyes-Hernández et al., 2019).

**FIGURE 3:**
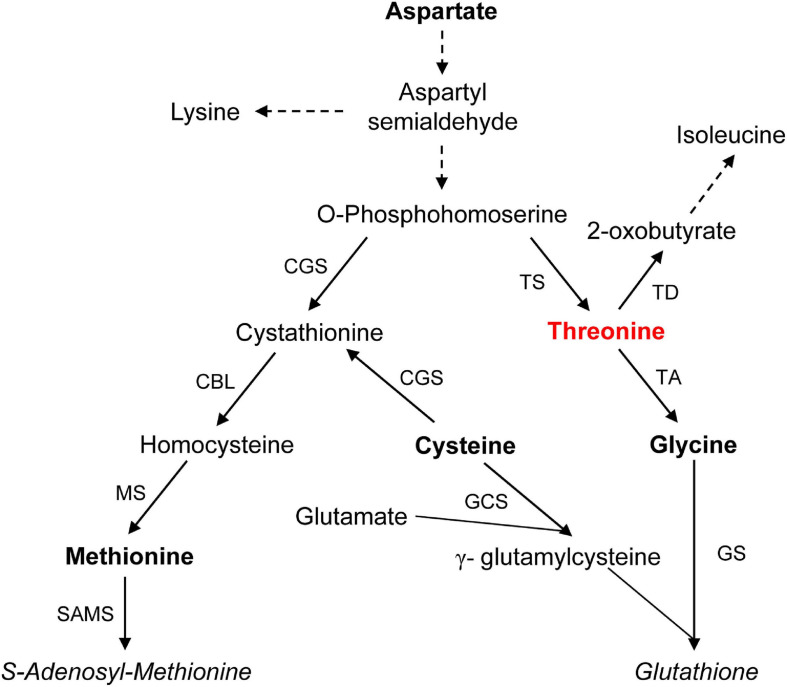
Aspartate-derived Thr synthesis and its catabolism and related Met and glutathione synthesis in plants. Dashed lines indicate multiple enzymatic steps. Abbreviations: CBL, cystathionine β-lyase; CGS, cystathionine γ-synthase; GCS, γ-glutamylcysteine synthetase; GS, glutathione synthetase; MS, Met synthase; SAMS, *S*-adenosylmethionine synthase; TA, Thr aldolase; TD, Thr deaminase/dehydratase; and TS, Thr synthase. Based on Alscher (1989); Jander and Joshi (2009), and Kusano et al. (2010).

The first study that identified *A. thaliana MTO2*-deficient mutant, *mto2-1*, did not address the role of Thr in stem cell and the RAM maintenance (Bartlem et al., 2000). It came out that *mto2-1* is allelic to *mko1*, and so it was renamed to *mto2-2* (Reyes-Hernández et al., 2019). In the Thr synthesis pathway, *O*-phosphohomoserine (Giovanelli et al., 1984; Bartlem et al., 2000; Amir et al., 2002) is a common precursor for synthesis of Thr and Met, and if a mutation blocks Thr synthesis, high Met level is expected (Figure 3). Indeed, one order of magnitude higher Met level is found in *mto2* mutants (Bartlem et al., 2000; Kusano et al., 2010; Reyes-Hernández et al., 2019). It was shown that high endogenous Met level does not cause the RAM exhaustion (Reyes-Hernández et al., 2019) and when Thr is added exogenously, *mto2* mutants recover normal root growth (Bartlem et al., 2000; Reyes-Hernández et al., 2019). The first cells whose activity becomes reestablished in presence of Thr are of the QC and stem cells and subsequently the transit-amplifying domain is rebuilt (Reyes-Hernández et al., 2019). At the same time, when the RAM exhaustion is taking place in *mto2-2*, usually quiescent QC cells start to divide and stem cells eventually differentiate (Hernández-Barrera et al., 2011; Reyes-Hernández et al., 2019). Therefore, Thr appears to be critical for stem cell activity and the RAM maintenance.

Resemblance of importance of Thr for stem cell maintenance in plants and animals is intriguing. The *mto2* mutant phenotype and recovery of the RAM in presence of Thr suggests that the Thr-dependent pathways represents a novel regulatory pathway essential for stem cell activity in plants (Figure 2). How it is related to the known regulatory pathways involved in stem cell activity is still an open question. The fact that *SCR*, *WOX5* (Hernández-Barrera et al., 2011), and *PLT1* (unpublished data) promoter activity is found in the *mto2-2* root apex even when RAM is exhausted suggests that Thr-dependence of the RAM maintenance represents a pathway independent of the SCR-SHR, PLT, and WOX5. Apparently, it is also independent of auxin as auxin response at transcriptional level is still detected in *mto2-2* seedlings (Hernández-Barrera et al., 2011).

Interestingly, THREONIN SYNTHASE 2 (AT1G72810) may compensate TS1 mutation in *mto2* mutant. SAM acts an allosteric regulator of enzymatic activity of the TS1 (Curien et al., 1998; Mas-Droux et al., 2006) and TS2 (Morneau, 2014). At low SAM levels, TS2 has a two orders of magnitude lower activity compared to that of TS1. However, when SAM level is high, TS2 activity is similar to that of TS1 (Curien et al., 2009). Whether *MTO2* role in stem cell maintenance is specific for these cells, how it may interact with TS2 activity, where exactly in the RAM *MTO2* expression is critical are all open questions. The mechanism for Thr-dependent stem cell function in plants is not known. Next, we consider possible ways in which Thr metabolism may regulate stem cell activity in plants.

## Possible Mechanisms of Thr-Dependent Plant Stem Cell Maintenance

The Thr level in animal systems is critical for maintenance of stemness, and in mES cells it is maintained at a very low level. During transition to differentiation, the Thr level increases (Wang et al., 2009). Increased Thr level during differentiation of mES cells corresponds well with increased Thr level in *mto2-2* roots compared to wild type; in the mutant, the RAM is developmentally lost, and all cells become differentiated (Reyes-Hernández et al., 2019). This seems to be paradoxical as lack of TS1 function should result in less Thr. However, the fact that exogenous Thr rescues the root growth in *mko1* (Reyes-Hernández et al., 2019) suggests unequal distribution (compartmentalization) of free Thr along the root and its deficiency in the root tip. Thus, similar to animal systems, a very low but available free Thr likely is required for the RAM stem cell activity. Therefore, a fine balance between Thr synthesis and its catabolism should be of great importance for stem cell function.

### Threonine Catabolism in Plants

Two groups of enzymes participate in Thr catabolism: Thr aldolases and a deaminase/dehydratase. THREONINE ALDOLASE1 (THA1) and THA2 are cytosolic enzymes that catalyze a conversion of Thr to glycine (Gly) and acetaldehyde (Jander et al., 2004; Joshi et al., 2006), Figure 3. As expected, *tha1* loss-of function mutants show up to 20 times higher Thr level (Jander et al., 2004); some *tha2* mutant alleles are lethal presumably due to a very high Thr level (Joshi et al., 2006). Contrary to animal systems, in plants Gly is not converted back to Thr (Joshi et al., 2006). A single Thr deaminase/dehydratase is plastid-localized enzyme, that converts Thr to 2-oxobutyrate, a precursor of isoleucine; it is encoded by *O-METHYLTHREONINE RESISTANT 1* (*OMR1*) gene (Joshi et al., 2006; Jander and Joshi, 2009). Importantly, all three genes involved in Thr catabolism show relatively high expression level in the RAM (Joshi et al., 2006; Brady et al., 2007; Winter et al., 2007). Based on rescuing of double *tha1tha2* mutants achieved by *OMR1* overexpression, Jander and Joshi (2009) concluded that Thr to Gly conversion “is not essential for *A. thaliana*” (see also Joshi et al., 2006). Nevertheless, it was shown that in *tha1* mutants Gly content is significantly decreased (Joshi et al., 2006) showing importance of Thr aldolases to maintain the Gly pool. In plants, Gly is also produced in photosynthetic tissues through photorespiration (Buchanan et al., 2015). It is highly probable that in such heterotrophic tissues as the root tip, where photorespiration does not take place (Walton and Woolhouse, 1986), conversion of Thr to Gly by Thr aldolase is a main source of Gly. This amino acid also participates in synthesis of glutathione (Figure 3), which in turn is critical for Redox state and stem cell activity in the RAM (Cheng et al., 1995; Vernoux et al., 2000; Considine and Foyer, 2021). Considering that Gly is essential for 1C metabolism, Thr aldolase activity should be of special importance in heterotrophic plant tissues 1C metabolism. Thus, a Thr-dependent mechanism of stem cell maintenance in plants could have a component similar to that in animals.

### 1C Metabolism in Root Stem Cells May Be Thr Dependent

In photosynthetic plant cells, Gly is oxidized during photorespiration by mitochondrial glycine decarboxylase complex (GDC, Bourguignon et al., 1988). It has been shown that GDC is also active in heterotrophic tissues (Mouillon et al., 1999). In folate-dependent 1C metabolism, GDC converts Gly and THF to 5,10-CH_2_-THF (methylenetetrahydrofolate), carbon dioxide, and ammonia. Subsequently, 5,10-CH_2_-THF and a second molecule of Gly are converted to Ser and tetrahydrofolate (THF) by serine hydroxymethyltransferase (SHMT); this reaction is fully and easily reversible (Mouillon et al., 1999; Douce et al., 2001; Hanson and Roje, 2001; Hanson and Gregory, 2011). The 1C derivatives of THF are interconverted between different oxidation states, ranging from 10-formyl-THF (most oxidized) to 5-methyl-THF (most reduced; Figure 2) within mitochondria and other subcellular compartments. 5-Methyl-THF is used to convert homocysteine to methionine, which is further converted to SAM, the biochemical methyl donor (Roje et al., 1999; Hanson et al., 2000; Roje, 2006). 10-formyl-THF is used in two transformylation reactions for *de novo* purine biosynthesis (Zrenner et al., 2006) and 5,10-CH_2_-THF is used for thymidylate synthesis (Lazar et al., 1993).

In heterotrophic plant tissues, Ser replaces Gly as the main source of 1C units, and catabolism of Gly is about threefold faster than that of Ser (Mouillon et al., 1999). Thus, the Ser/Gly ratio can be two to 10 times greater in the roots than in the shoots (Wulfert and Krueger, 2018; Reyes-Hernández et al., 2019). If Thr catabolism is essential for 1C metabolism, then the Ser/Gly ratio should be affected in *mto2* mutant roots. Indeed, it is 1.8 greater in the mutant compared with wild-type roots (Reyes-Hernández et al., 2019), supporting the possibility that a mutation in *MTO2* affects 1C metabolism.

In non-photosynthetic cells, other than root tip, it is estimated that a single mitochondrion produces 61,989 molecules of serine and THF per second and that SHMT2 is 102 times more abundant than SHMT1 compared to photosynthetic tissue (Fuchs et al., 2020). This is consistent with moderate *SHMT2, SHMT3 (AT4G32520), SHMT4 (AT4G13930)*, and almost non-existent *SHMT1* gene expression within the RAM (Brady et al., 2007; Winter et al., 2007). Interestingly, in accordance with the same transcriptomic analysis, *SHMT7* (*AT1G36370*) has about 5- to 10-fold higher expression level in the columella stem cells compared to surrounding RAM tissues. The *SHMT7* gene is a non-canonical SHMT but has a role in SAM accumulation and DNA methylation specifically in the root (Huang et al., 2016). These data together support the conclusion that Thr catabolism in the root tip is likely a critical source of Gly and that 1C metabolism in stem cells may be Thr dependent.

### Possible Consequences of Hypothesized Thr-Dependent 1C Metabolism in Stem Cells

If the proposed Thr-dependent 1C-metabolism in stem cells is correct, it should be possible to find 1C-metabolism-related mutants with a phenotype similar to that of *mto2-2*. Indeed, mutants in plastidial *FOLYPOLYGUTAMATE SYNTHETASE1* (*FPGS1*) show short root phenotype (Srivastava et al., 2011; Reyes-Hernández et al., 2014). One such mutant, *mko2*, similar to *mko1* (*mto2-2*) has almost identical phenotypic abnormalities: despite normal RAM formation during embryogenesis, *mko2* exhibits loss of stem cell activity and complete RAM exhaustion post-germination and activation of cell proliferation in the QC during the RAM exhaustion and cell differentiation at the root tip (Hernández-Barrera et al., 2011; Reyes-Hernández et al., 2014, 2019). These studies also demonstrate surprisingly similar expression of the RAM maintenance markers, such as WOX5, PLT, and SCR that are maintained despite the RAM exhaustion. This comparison of *mko1* (*mto2-2*) and *mko2* supports the hypothesis that Thr-dependent 1C-metabolism is needed for stem cell maintenance.

A link between metabolism, epigenetic modifications, and plant stem cell maintenance became more evident during the last two decades (Takatsuka and Umeda, 2015; Lindermayr et al., 2020; Samo et al., 2021). It has been shown that a mutation in FPGS1, and a disbalance in folate metabolism negatively affects DNA and histone methylation (Zhou et al., 2013; Groth et al., 2016). Convergently, changes in Thr metabolism in *mto2-2* could affect 1C metabolism not only through Gly-Ser balance but also through increased Met synthesis which is a precursor of SAM both in plants and animals. SAM serves as the substrate for methyltransferase catalyzed DNA and histone methylation (Loenen, 2006; Vaillant and Paszkowski, 2007; Feng et al., 2010; Law and Jacobsen, 2010). Similarly, a mutation in *CYSTATHIONINE BETA-LYASE* (*CBL*), a plastidial enzyme which catalyzes the penultimate step of *de novo* Met synthesis (see Figure 2), leads to reduced levels of global H3K4me3 histone modification and DNA methylation in the root (Liu et al., 2019). In the *cbl* mutant, H3K4me3 trimethylation is decreased at *PLT1* and *PLT2* genes and respective gene expression is decreased in the RAM resulting in a significant decrease in RAM length (Liu et al., 2019). For all of these reasons, 1C metabolism in RAM stem cells likely can be linked to Thr metabolism and the resultant epigenetic control of stem cell activity, as is the case for H3K4me3 formation in mammalian ES cells (reviewed in Van Winkle and Ryznar, 2019b).

## Conclusion

### Similarities and Differences Between Plant and Animal Stem Cell Thr Metabolism

As discussed, plants not only synthesize Thr but also maintain Thr balance, whereas trophoblast cells likely provide Thr to ES progenitor cells in blastocysts through protein hydrolysis. Nevertheless, Thr catabolism is involved in 1C metabolism of both plants and animals. In both kingdoms, Thr and 1C metabolism may control stem cell function *via* H3K4 histone methylation. Despite possible similarities between plant and animal stem cell metabolism, cell cycle-related phenomena are very different. The cell cycle duration in mouse ES cells is only 5 h (Wang et al., 2009). In the root apical meristem, the shortest cycle time is found in the transit-amplifying domain while the intermediate and longest cycle times are in stem cells and in the QC (Rahni and Birnbaum, 2019), respectively. Nevertheless, Thr could be critical not only for stem cell function in the RAM but also in the transit-amplifying domain.

Moreover, Thr aldolases have been found in various organisms including bacteria, fungi, mammals, and plants (Jander et al., 2004; Edgar, 2005; Liu et al., 2015). In plants, they play a major role in Thr catabolism. These enzymes are found in the plant cytosol (van Maris et al., 2003; Jander et al., 2004), whereas they are localized in vertebrate mitochondria (Edgar, 2005). Thr aldolase is a non-transcribed pseudogene in humans, but in mouse embryos the Thr aldolase mRNA level is similar to that of TDH (Edgar, 2005). Thus, Thr aldolases in vertebrates may function in cooperation with TDHs, and more research is needed to establish their role in stem cell maintenance.

### Perspectives

In both plants and animals, Thr metabolism is likely critical for stem cell function. As for plant stem cell activity and regulation (De Tullio et al., 2010), animal stem cell maintenance is also linked to specific redox states that may involve Thr metabolism (Wang et al., 2013). In glutathione-treated 8–16 cell bovine embryos, for example, the Thr pathway becomes one of the most enriched (Guo et al., 2020), suggesting a link between Thr metabolism and redox regulation. Moreover, Thr derived Gly and other Thr metabolites serve as precursor of glutathione (Tian et al., 2019). In plants, Thr metabolism could also be related to glutathione synthesis as Thr synthesis in plastids competes with Met production, which uses Cys as a precursor (Figure 3). Glutathione biosynthesis begins in plastids with participation of gamma-glutamylcysteine synthetase which is considered a rate-limiting enzyme, and Cys availability in plastids is known to affect glutathione, Met, Thr, and protein syntheses (Ravanel et al., 1998; Vernoux et al., 2000; Nguyen et al., 2012; Speiser et al., 2018). Thus, a disruption of Thr metabolism has the potential to impact the plastidic Cys pool and affects the Gly and Ser pools. Moreover, disruption in glutathione biosynthesis induces the RAM exhaustion (Cheng et al., 1995; Vernoux et al., 2000). These Thr metabolic crossroads with glutathione synthesis make it appealing to investigate further how exactly Thr metabolism is linked to the redox state maintenance in stem cells in both kingdoms.

The role of Thr in the shoot apical meristem maintenance in plants has not been yet reported. The fact that the Thr content in the *Asparagus officinalis* shoot meristem and neighboring tissues in the apex is the lowest compared with the more basal shoot portions (Creydt and Fischer, 2020) is consistent with expected low Thr level in the RAM. Thr biosensors could help in the analysis of free Thr distribution at a tissue and cellular level. Understanding of the role of Thr in plant systems is behind similar studies in animal systems. Many questions are still open. For example, in mammalian ES cells signaling may occur via a plasma membrane Thr transporter (transceptor) and subsequent mTOR activation (Ryu and Han, 2011; Van Winkle and Ryznar, 2019a). Such transport could also help to maintain pluripotency in plant stem cells. Amino acid transceptors likely exist in plants, but their identification remains an active field of investigation (Dinkeloo et al., 2018). Whether Thr in plants may function as a signaling molecule, and whether it is perceived by TOR signaling system which show some conserved elements in plants and animals (Shi et al., 2018; Burkart and Brandizzi, 2020) is yet to be established. How Thr transport could be involved in stem cells function, what are the mechanisms of fine-tuning of Thr level, and what is the concrete link between Thr metabolism and gene regulation at an epigenetic level, all are intriguing questions that hopefully soon will be addressed.

As all plant organs develop from meristems, their function is principal for biomass production and yield. Thus, understanding the meristem function and its dependence on Thr metabolism can have potentially applied significance. Based on Thr role in stem cell maintenance in animal systems, we proposed here the Thr-catabolism-related mechanism of stem cell maintence in plants that provides a link between energy status, epigenetic changes, and stem cell activity. This link should be further explored.

The present analysis also suggests that while Thr is critical in angiosperm plant and mammal ES cell maintenance, it also may have a similar role in other plant and animal phyla. It is highly probable that Thr can also be critical in stem cell maintenance in Bryophytes, Lycophytes and other than Angiosperms Euphyllophytes plant species. Similarly, Thr can be essential for stem cell maintenance in other mammal animal phyla. Our interkingdom comparison also suggests a universality of Thr as a stem cell factor and, therefore, predicts that not only ES cell state but also stem cell postembryonic stages of animal development may depend on Thr.

### General Conclusions

(1) Our analysis demonstrates clearly that Thr is a critical amino acid involved in control of stem cell maintenance in both plants and animals, implying the existence of ancestral mechanisms to control stemness that emerged at the origin of multicellularity. The exact mechanisms of Thr dependence in maintenance of stemness in plants are not yet known, but existing metabolic fluxes suggest that at least part of the mechanisms can be related to Thr catabolism. Importantly, in both kingdoms, this control can operate through 1C metabolism that provides intermediate metabolites involved in epigenetic control, particularly H3K4 histone methylation, important for stem cell maintenance.
(2) Stem cell state depends on redox balance. Our data support the conclusion that, in both kingdoms, Thr metabolism is linked to factors involved in maintenance and regulation of the redox state, particularly, to glutathione synthesis. Future research should uncover details of this link.
(3) In animals, a permanent but low level of Thr in stem cells is essential for maintenance of stemness, and TDH is the main player in this control. Our analysis suggests that like in plant cells, animal aldolases coordinate with TDH to finely regulate Thr concentration in stem cells.
(4) In animals, the role of Thr in stem cell maintenance is known currently only for mammals. We propose that, if Thr dependence of stemness properties is a universal feature, as in plants and mammals, then Thr involvement in stemness in mammals functions beyond ES cells to stem cells at all stages of development. Additionally, it is highly probable that Thr plays a role in stemness also in other animal kingdom phyla.
(5) In plants, many metabolites function as signaling molecules. Signaling function of Thr in plants are not yet shown. If Thr is also a signaling molecule, systems similar to amino acid transceptors in animals are awaiting their research in plants. Our review suggests that a network of transcription factors involved in maintenance of meristem activity in plants should operate in coordination with a Thr-dependent pathway. It is highly probable that this coordination could be functional *via* mechanisms related to perception and signaling of Thr, an amino acid vital for maintenance of stem cell activity in plants.

## Author Contributions

JD and LV: conceptualization. DS, LV, RD, and JD: investigation, writing (review and editing), and visualization. DS and JD: writing (original draft). JD: funding acquisition and supervision. All authors participated in the editorial improvement of the text and approved the final manuscript.

## Conflict of Interest

The authors declare that the research was conducted in the absence of any commercial or financial relationships that could be construed as a potential conflict of interest.

## Publisher’s Note

All claims expressed in this article are solely those of the authors and do not necessarily represent those of their affiliated organizations, or those of the publisher, the editors and the reviewers. Any product that may be evaluated in this article, or claim that may be made by its manufacturer, is not guaranteed or endorsed by the publisher.
